# Optimal Management for Stage IVB Endometrial Cancer: A Systematic Review

**DOI:** 10.3390/cancers15215123

**Published:** 2023-10-24

**Authors:** Vito Andrea Capozzi, Elisa Scarpelli, Alessandra De Finis, Isabella Rotondella, Davide Scebba, Asya Gallinelli, Carlotta Montrucchio, Giulia Martignon, Martina Leotta, Tullio Ghi, Roberto Berretta

**Affiliations:** Department of Obstetrics and Gynecology, University of Parma, 43125 Parma, Italy

**Keywords:** endometrial cancer, stage IVB endometrial cancer, advanced-stage endometrial cancer, debulking surgery, adjuvant treatment

## Abstract

**Simple Summary:**

Stage IVB endometrial cancer, as defined in the 2009 edition of the FIGO staging system, encompasses a diverse group of patients with a wide-ranging distribution of disease, including intra- and extra-abdominal metastasis. The presence of peritoneal carcinomatosis and/or distant or parenchymal metastasis complicates the establishment of definitive recommendations regarding the optimal primary treatment and adjuvant therapy. Current guidelines advocate for primary cytoreductive surgery when feasible, while there are no stringent indications for adjuvant treatment. Meanwhile, neo-adjuvant chemotherapy is increasingly employed, primarily due to the growing reassuring evidence in ovarian cancer. However, evidence in the context of advanced-stage endometrial cancer mainly derives from retrospective case series. A systematic review of the current literature may assist in identifying which factors should be considered when determining the optimal management for stage IVB endometrial cancer patients.

**Abstract:**

(1) Background: Endometrial cancer (EC) is a common gynecological malignancy, often diagnosed at an early stage with a high overall survival rate. Surgical treatment is the primary approach, guided by pathological and molecular characteristics. Stage IVB EC, characterized by intra and/or extra-abdominal metastasis, presents a significant challenge with no clear consensus on optimal management. (2) Methods: A systematic literature review was conducted from January to May 2023, covering studies from 2000 to 2023. Eligible studies included retrospective case series, prospective trials, and randomized clinical trials. (3) Results: Of 116 studies identified, 21 were deemed relevant: 7 on primary surgery, 10 on neoadjuvant chemotherapy (NACT), and 4 on adjuvant treatment. Notably, the impact of residual tumor after primary surgery was a critical factor affecting survival. The use of NACT followed by interval debulking surgery showed promise, particularly in cases deemed unresectable. Adjuvant treatment, combining radiotherapy and chemotherapy, demonstrated improved survival but lacked consensus regarding its role. (4) Conclusions: Stage IVB EC poses a complex challenge with limited evidence to guide management. Optimal cytoreduction remains crucial, and NACT should be considered for unresectable cases. Multimodality adjuvant therapy may benefit patients, even with disease spread beyond the pelvis. Future advances in molecular classification and targeted therapies are expected to enhance treatment strategies.

## 1. Introduction

Endometrial cancer (EC) is the most common gynecological malignancy and it is mostly diagnosed at an early stage. Overall survival (OS) for early stage EC patients reaches 95% at 5 years [1]. According to international guidelines, surgical treatment consists of total hysterectomy, bilateral salpingo-oophorectomy, and nodal staging through either sentinel node biopsy or retroperitoneal lymphadenectomy [2]. Pathologic characteristics, immunohistochemistry, and molecular features define the prognostic risk and the need for adjuvant therapy [2,3,4]. External beam radiotherapy, brachytherapy, and chemotherapy are the mainstay of adjuvant treatment, used in different combinations depending on the tumor’s prognostic risk and patients’ performance status. However, ongoing randomized clinical trials (RCT) predict personalized adjuvant therapy based on tumor molecular classifications [5].

Following the 2009 edition of the FIGO staging system, EC presents at stage IVB in 3–13% of cases and was defined as the presence of intra- and/or extra-abdominal metastasis [6]. In this case, patients show a poor prognosis, with a 10–20% 5-year overall survival rate [7]. Given the rarity of this advanced stage and the heterogeneity of patients included, optimal management is not unanimous [8,9]. Furthermore, strong recommendations are lacking due to the absence of high-quality evidence from the literature. Reported survival benefits seem to depend on pathological features, metastasis locations, primary treatment, and adjuvant therapy [10]. However, interesting perspectives seem to be emerging also in this peculiar set of patients. The improvement of our knowledge of tumor biology, and the availability of targeted therapy, are leading to promising therapeutic results [11,12]. Moreover, the 2023 FIGO staging system for EC provides a more precise stratification of patients.

Current international guidelines recommend upfront surgery when complete cytoreduction is feasible. In cases of unresectable disease at presentation, definitive local radiotherapy followed by adjuvant chemotherapy should be considered. Alternatively, a neoadjuvant treatment approach and subsequent surgery may be contemplated.

However, a clear decision algorithm has not been established yet [2], and a consensus on adjuvant treatment is lacking in this context.

This manuscript aims to provide an overview of the current evidence for the optimal management of FIGO stage IVB EC. Specifically, options for primary treatment, benefits, and modalities of adjuvant treatment according to the most updated literature were analyzed.

## 2. Materials and Methods

A systematic review of the literature was performed in double-blind by two authors (V.A.C. and E.S.). The analysis was conducted from January 2023 to May 2023 and included published literature from 2000 to 2023. A third author (R.B.) checked the selected articles. Research on Pubmed, Web of Science, and Scopus was carried out. The search utilized a set of specific keywords and keyword combinations, including “endometrial cancer” and “stage IVB,” “endometrial cancer” and “advanced stage disease,” “endometrial cancer” and/or “peritoneal carcinomatosis,” “endometrial cancer” and/or “peritoneal metastasis,” “stage IVB endometrial cancer” and “primary surgery” or “neoadjuvant chemotherapy,” and “stage IVB endometrial cancer” and “adjuvant treatment,” “radi-otherapy,” “chemotherapy,” or “chemo-radiotherapy.” This search strategy aimed to identify and select the most pertinent articles for the systematic review.

The agreement about potential relevance was reached by consensus of the researchers and according to PRISMA statement guidelines [13]. After the first selection, the authors evaluated the full-text copies of selected papers and separately extracted relevant data regarding study characteristics and outcomes. All references were analyzed to evaluate additional eligible studies. Retrospective case series, prospective trials, and RCTs were included in the research. Studies considered not in line with the purpose of the study, case reports, redundant studies, not full-text manuscripts, and articles not in the English language were excluded.

## 3. Results

**Figure 1:**
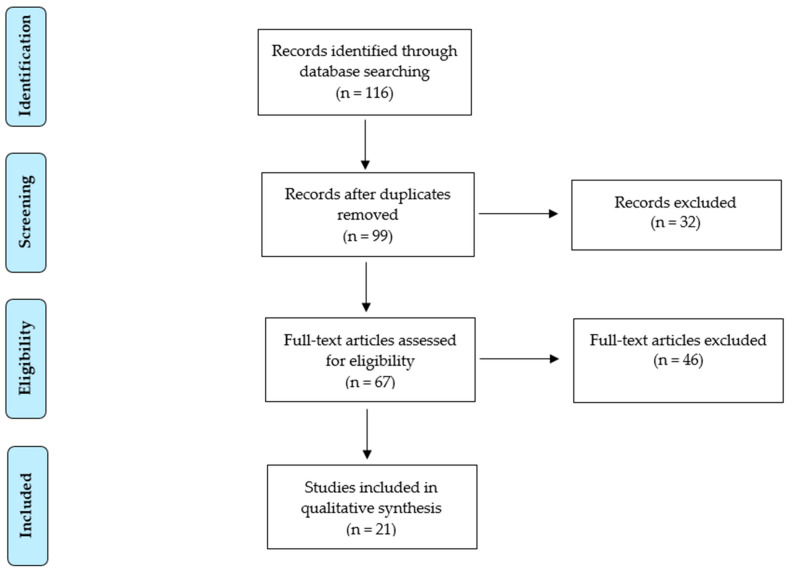
PRISMA flow chart.

The electronic database search provided a total of 116 studies, of which 17 duplicates, 25 case reports, 7 studies not in the English language, and 46 works not fitting the review scope were excluded from the analysis. The study selection flowchart is shown in Figure 1.

A total of 21 studies were considered eligible for the study: 7 studies regarding primary surgery and residual tumor (Table S1), 10 studies on neoadjuvant chemotherapy (NACT) (Table S2), and 4 studies about adjuvant treatment (Table S3). All included studies are retrospective; further details on the study design are provided in the tables.

To make reading easier, the main findings are reported in sections as follows: prognostic impact of residual tumor at primary surgery; use of neoadjuvant chemotherapy and interval debulking surgery; and adjuvant treatment. At the close of each section, a comprehensive synthesis of available the evidence is provided, and an assessment of the quality of the evidence is conducted, based on the Grading of Recommendations Assessment, Development, and Evaluation (GRADE) system [14].

## 4. Discussion

### 4.1. Impact of Residual Tumor at Cytoreductive Surgery

Traditionally, primary debulking surgery (PDS) is the standard of care in stage IVB EC. Despite the poor prognosis of the disease, indeed, surgery is known to be beneficial in this set of patients [2]. However, evidence about surgery in stage IVB EC is limited due to the lack of randomized clinical trials. Different authors investigated the value of residual tumor volume in terms of OS and PFS, and the impact of other prognostic factors.

In 2000, Bristow et al. conducted a retrospective review of 65 patients with stage IVB EC including 9 patients with extra-abdominal metastasis [15]. The rate of optimally debulked patients was 55.4%. The median OS was 14.8 months. The study showed that patients optimally debulked had a median OS of 34.3 months while patients with a residual tumor (RT) >1 cm after surgery had a median survival of 11 months. Furthermore, patient’s performance status and age under 58 years were identified as prognostic factors affecting survival on multivariable analysis.

In line with these observations, in a case series on 37 patients receiving primary cytoreductive surgery, Ayhan et al. reported a median survival of 25 months for patients optimally debulked (<1 cm), and 48 months survival when considering patients reaching complete cytoreduction [16]. In multivariable analysis, the absence of extra-abdominal metastasis, optimal cytoreduction, and multimodality adjuvant treatment (see Section 4.3) were revealed as significant factors for improved survival outcomes.

The prognostic value of optimal cytoreductive surgery was also recently demonstrated by Haight et al. in a retrospective study including 88 patients [17]. Among these, 63 patients underwent primary surgery, and 51 had RT less than 1 cm. The study revealed that residual tumor size had a significant prognostic impact along with pelvic-confined disease.

Regarding a specific histology type, Lee et al. conducted a retrospective study including only patients with serous histology and intra-abdominal metastasis [18]. In total, 48 patients were included, and 36 (75%) had a residual tumor <1 cm. Comprehensive 5-year overall survival was 19% and 12 months of progression-free survival was reported. In this study also, optimal cytoreduction and adjuvant chemotherapy were identified as prognostic factors for long-term survival.

Some authors raised concerns about the inclusion of extra-abdominal disease in the 2009 FIGO IVB stage. This aspect makes observations about the best treatment arduous to elucidate and generalize. Therefore, different studies investigated the role of primary cytoreduction in the presence of distant metastasis.

In 2012, Eto et al. published a large multicentric retrospective study including 248 patients with EC stage IVB who underwent primary surgery [19]. In this study, 93 patients had extra-abdominal disease (37.5%), mainly with lung metastasis (*n* = 49). Complete resection was achieved in 101 patients (40.7%). The study showed 24 months of overall survival in the entire population and 48 months of survival in patients with no residual disease. At multivariate analysis, performance status, histology and grade, adjuvant treatment, and intra-abdominal residual disease were independent prognostic factors, even in the presence of extra-abdominal disease.

The favorable prognostic impact of primary cytoreduction even in cases of distant metastasis, was reported also by Ueda et al. [20]. In a retrospective analysis of 30 patients, a significant benefit in prognosis was reported for residual disease <2 cm vs. >2 cm, both in patients with intra- and extra-abdominal metastasis. The median OS was comparable between patients with intra- and extra-abdominal metastasis undergoing cytoreductive surgery (20 vs. 22 months), while optimal cytoreduction significantly affected overall and progression-free survival in both groups.

In light of these results, a resemblance between advanced ovarian cancer and stage IVB EC has been hypothesized by some authors.

Interestingly, Landrum et al. analyzed 165 patients (55 endometrial cancer and 110 ovarian cancer) to compare endometrial and ovarian cancer survival outcomes after matching for age and residual disease [21]. The study reported a 2-year survival rate of 52% and progression-free survival of 13 months in EC patients. Furthermore, the authors reported better ovarian cancer survival outcomes compared to endometrial cancer (76% vs. 52% 2-year overall survival and 20 vs. 13 months progression-free survival, respectively). Nevertheless, the study also confirmed that residual tumor <1 cm persisted as a predictor of better survival in both endometrial and ovarian cancer patients.

In summary, the prognostic significance of residual disease after debulking surgery for stage IVB EC appears to reflect the evidence observed in ovarian cancer. However, the overall prognosis between the two types of cancer seems to differ. Complete or optimal cytoreduction is unanimously recognized as a leading prognostic factor in stage IVB EC. The prognostic benefit of at least optimal cytoreduction seems to be maintained, independently from the histology and extra-abdominal spread of the disease [19,20]. However, histology and extra-abdominal disease impact patients’ survival. The available evidence emerged from retrospective studies with a limited number of patients.

***Summary of the available evidence:*** Optimal cytoreduction is a key prognostic factor in stage IVB EC, independent of histology and extra-abdominal spread (*level of evidence: low*).

### 4.2. Use of Neoadjuvant Chemotherapy in Stage IVB Endometrial Cancer Patients

As previously discussed, cytoreductive surgery with complete removal of macroscopic residual tumor is unanimously recognized as a good prognostic factor, even in advanced EC. However, there is growing evidence borrowed from ovarian cancer suggesting that chemotherapy before surgery may also be considered. Following the ESGO guidelines, neoadjuvant chemotherapy (NACT) could be assessed in cases of unresectable disease and/or in patients unfit for extensive surgery [2].

Three randomized clinical trials (EORTC, CHORUS, and the SCORPION trial) in ovarian cancer showed that interval debulking surgery yields comparable survival outcomes to primary debulking surgery [22,23,24]. Moreover, the TRUST trial will provide elucidating results in this set of patients [25]. In addition, primary debulking surgery is associated with high postoperative morbidity affecting up to 50% of patients also in the context of EC cases [15,26]. Nevertheless, NACT is not yet the standard treatment for advanced-stage EC. Therefore, in the absence of strong evidence, some authors investigated the role of NACT in stage IVB EC [27].

In 2009, Vandenput et al. investigated the role of NACT followed by interval debulking surgery (IDS) in stage IVB EC with intra-abdominal disease [28]. A total of 30 patients were enrolled and received 3–4 cycles of neoadjuvant chemotherapy with carboplatin-paclitaxel. In total 24 patients underwent IDS, and all patients were optimally debulked, 22 (80%) with no residual disease.

In 2013, Eto et al. compared primary surgery with preoperative chemotherapy and subsequent IDS in a retrospective analysis of 426 patients with stage IVB EC [29]. The median OS was significantly higher in the primary surgery group than in the chemotherapy group (21 months vs. 12 months, *p* = 0.0001). Primary surgery patients had a better performance status and a lower rate of clinical intra-abdominal metastasis. However, when considering only patients who actually underwent surgery after chemotherapy (59 cases), overall survival was comparable between the two groups (primary surgery vs. primary chemotherapy). Patients who received preoperative chemotherapy had a higher rate of optimal cytoreduction (57% vs. 45%) compared to the primary surgery group. However, this difference was not statistically significant. Interestingly, this study also included patients who underwent only palliative care or chemotherapy without subsequent surgery, and as expected overall survival was extremely poor in these patients. Finally, in this study, extra-abdominal metastasis did not impair patients’ prognosis at multivariate analysis. Therefore, the authors concluded that surgery could be also considered in patients with extra-abdominal disease and chemotherapy should be the first approach in patients judged unresectable at primary surgery without detrimental effect on survival.

Recently, Zhangh and Kanno et al., confirmed these results. Both authors reported that the association of cytoreductive surgery and chemotherapy, irrespective of the timing, revealed improved oncological outcomes in all stage IVB EC patients, even in the presence of long-distance metastasis when compared to the chemotherapy-only group [30,31].

In line with the study conducted by Eto et al., Tobias et al. published a large cohort study based on the National Cancer Database including 4890 patients with EC at stage IV, of which 3922 were stage IVB cases [32]. The objective of the study was to compare the survival of patients receiving NACT vs. primary surgery. Intention-to-treat analysis and per-protocol analysis were conducted, and in both cases, the results showed that NACT was associated with higher survival rates during the first 3–8 months after primary treatment, but after this period, NACT patients showed increased mortality compared to the surgery group.

A similar trend was reported by Jooya et al., in an analysis of 5505 stage IVB EC patients receiving primary chemotherapy or surgery between 2010–2018 [33]. The study showed comparable oncological outcomes between the two groups (25 months overall survival in the NACT group vs. 26 months in the surgery group). However, excluding patients with distant metastases (lung, liver, bones, and brain), the NACT group was associated with decreased overall survival compared to the primary surgery group (HR = 1.2, 95% CI 1.05–1.36).

Considering previous results on preoperative chemotherapy and serous ovarian cancer treatment, and pathologic and prognostic similarities between serous ovarian cancer and serous EC, some authors considered this histology in particular [34,35,36].

In a retrospective pilot study by Wilkinson-Ryan et al., 44 patients with stage IV serous EC were analyzed [35]. In total, 10 patients underwent primary chemotherapy with carboplatin and taxanes (3–8 cycles), and 34 patients underwent primary surgery. Overall and disease-free survival did not differ between the two groups, even if a higher proportion of NACT patients achieved complete cytoreduction (70% vs. 32%), and almost 18% of patients undergoing primary surgery had residual tumors >1 cm. Moreover, the primary chemotherapy group had a shorter length of hospital stay and operative time compared to the primary surgery group.

In line with these results, Bogani et al. conducted a propensity match score analysis including 15 Stage IV patients who received neoadjuvant chemotherapy followed by surgery, and 19 patients who underwent primary cytoreductive surgery, all diagnosed with a stage IVB serous EC [34]. Twelve patients received 3–6 cycles of carboplatin-paclitaxel, and the other three patients received 3 cycles of paclitaxel, doxorubicin, and cisplatin. The median overall survival was 16.7 months in the IDS group and 18 months in the primary surgery group, while disease-free survival was 12 and 15 months, respectively. In this study also, patients undergoing IDS experienced a minor length of hospital stay and duration of surgery with comparable postoperative morbidity compared to primary surgery patients.

In conclusion, primary surgery with optimal cytoreduction should be the preferred option in IVB EC. Primary chemotherapy and subsequent surgery should be considered in nonresectable disease. Preoperative chemotherapy could be a good option in serous histology. The presence of extra-abdominal disease should not be a sufficient reason to refer patients for NACT treatment.

Due to the rarity of the disease, there is a lack of strong evidence, and most observations are based on retrospective series without standardized protocols. The chemotherapy regimens and the number of cycles vary widely among the reported case series.

***Summary of the available evidence:*** Primary chemotherapy followed by surgery is an option for unresectable disease. Preoperative chemotherapy may be suitable for serous histology. The presence of extra-abdominal disease should not be the sole determinant of neoadjuvant chemotherapy (*level of evidence: low*).

### 4.3. Adjuvant Treatment in Stage IVB Endometrial Cancer

Adjuvant treatment after surgery for advanced-stage EC can be based on radiotherapy, chemotherapy, or a combination of both modalities [2].

Traditionally, whole abdominal radiotherapy or pelvic radiotherapy has been administered after surgery. However, despite good local disease control, distant relapses impair survival. On the other hand, pelvic recurrence rate is 20% if only chemotherapy is chosen [37]. Therefore, available data support the use of multimodality treatment.

The PORTEC 3 trial was an international, multicentre, randomized phase 3 trial, which aimed to investigate the survival benefit of adjuvant chemoradiotherapy vs. radiotherapy alone in high-risk EC, stage I-III. Chemo-radiotherapy superiority over pelvic radiotherapy alone was reported in terms of disease-free survival, especially in serous histology [38]. Furthermore, a post-hoc analysis including molecular classification confirmed these results in p53abn tumors [39]. Subsequently, the GOG258 study included stage III-IVA EC patients and reported that chemo- plus radiotherapy was superior to chemotherapy alone in local recurrence control, even in the absence of an absolute survival benefit. Indeed, chemotherapy alone is an option, but concurrent chemo- plus radiotherapy is still considered the gold standard adjuvant treatment in advanced EC FIGO stage [40]. Concerning the chemotherapy regimen, the GOG0209 study established carboplatin-paclitaxel as the preferred scheme, as it showed a non-inferiority with less toxicity compared to doxorubicin and cisplatin paclitaxel [41].

Since primary surgery is the preferred option in case of presumed resectability, current guidelines recommend an individualized approach with either radiotherapy and/or chemotherapy based on local recurrence and distant metastasis risk [2]. Furthermore, in the case of neoadjuvant chemotherapy and interval debulking surgery, adjuvant chemotherapy is a reasonable option.

As previously mentioned, Ayhan et al. published a retrospective study including 37 patients with stage IVB EC, of whom 6 patients had extra-abdominal metastasis (16.2%) [16]. Concerning adjuvant treatment, thirty patients received adjuvant treatment, and multimodality treatment was associated with prolonged survival. Median survival for patients receiving chemotherapy plus radiotherapy was 54 months, while overall survival was 15 and 13 months for radiotherapy and chemotherapy alone groups, respectively.

In 2016, Cirik et al. analyzed the survival advantage of chemotherapy over radiotherapy, testing the hypothesis that stage IVB EC with intra-abdominal extension may be considered a systemic disease [42]. A total of 65 patients were included, and complete cytoreduction was achieved in 54 cases (83.1%) and optimal cytoreduction in 4 cases (10.8%). In total, 62 patients (95.2%) received adjuvant therapy. The mean disease-free survival was 11 months, and the majority of the recurrences were outside the pelvis (*n* = 22, 78.5%). In multivariate analysis, adjuvant chemotherapy was a predictive factor of better overall and progression-free survival.

Recently, Barrington conducted a survival analysis based on large national registries (National Cancer Database and Surveillance, Epidemiology, and End Results), including 17,890 stage IV EC patients, of whom 16,133 EC patients were at stage IVB [43]. Per multivariable analysis, use of any type of radiotherapy, including external beam radiotherapy and vaginal brachytherapy, in addition to chemotherapy was associated with better overall survival compared to post-operative chemotherapy alone, except for clear cell histology. In particular, the most beneficial effect was reported in the case of chemotherapy plus external radiotherapy and brachytherapy. Anyway, national registers missed details on metastasis localizations; as a consequence, the author states that it is impossible to draw any definitive conclusion regarding the effect of radiotherapy on patients with extra-pelvic and/or extra-abdominal localizations vs. patients with pelvic-limited disease.

On the contrary, Haight et al., in the previously mentioned retrospective case series, registered also the effect of adjuvant therapy on PFS and OS, and concluded that multimodality treatment does not improve OS, but does lower local recurrence, thanks to the added effect of radiotherapy to systemic effect of chemotherapy [17]. Indeed, these results are in line with what is already known on chemo–radiotherapy vs. radiotherapy in high-risk EC.

In summary, chemotherapy, especially when combined with radiotherapy, improves survival outcomes. However, the generalizability of these results is limited because of the heterogeneity of stage IVB EC patients, particularly the presence of only intra-abdominal or also extra-abdominal metastasis. Currently, there is not enough evidence to exclude patients with stage IVB EC from radiotherapy adjuvant treatment, and chemotherapy should always be administered, since its beneficial effect on survival when combined with surgery is unanimously recognized.

***Summary of the available evidence:*** The integration of multimodal adjuvant therapies significantly improves survival rates for stage IVB EC patients. While radiotherapy is a valid consideration, adjuvant chemotherapy consistently leads to enhanced survival outcomes (*level of evidence: low*).

### 4.4. Final Considerations and Future Perspectives

Given the available literature, we can state that stage IVB EC, as defined in the 2009 edition of the FIGO staging system, is a rare entity with a life expectancy spanning from a few months to a few years and a short time to relapse [6]. However, definitive conclusions on the most beneficial treatment algorithm are arduous to define, due to the heterogeneity of histology, distribution of disease, and the variety of treatment protocols applied. While some authors have provided specific data on serous histology, there is a notable absence of specific evidence for other non-endometrioid histologies [18,26,28,34,35,36]. This gap results from the scarcity of cases and the lack of comprehensive data from retrospective studies.

Undoubtedly, achieving optimal cytoreduction during primary surgery is a significant factor to contemplate when deciding on initial management [44]. As specified by several authors, extra-abdominal metastasis does not completely invalidate the survival benefit derived from cytoreduction [19,20]; however, we may speculate that the location, number, and metastasis curability should be taken into account. Furthermore, the interpretation of studies may be misleading due to the widely heterogeneous localization of metastases and the fact that treatment of extra-abdominal metastasis is not always specified. In this regard, Eto et al. conducted a survival analysis on IVB EC patients considering the presence or absence of residual disease and its localization as intra-abdominal, extra-abdominal, or a combination of both. Interestingly, the authors revealed that in patients with intra-abdominal residual disease, a smaller size of residual disease was associated with longer OS. In contrast, the extra-abdominal residual disease was not related to OS (see Table S1) [19].

Neoadjuvant chemotherapy is a valuable alternative in patients ineligible for upfront cytoreductive surgery. Recently, its use has been increased by the encouraging results arising from randomized controlled trials in ovarian cancer patients [9,32]. Thus, the potential additional benefits of this approach in advanced-stage EC patients could be anticipated in the near future.

Concerning adjuvant treatment, there is not enough evidence to exclude the benefit of multimodality treatment, including external radiotherapy and brachytherapy in stage IVB EC [16,17,43]. While the role of chemotherapy seems undoubted [42] for distant disease, individual factors should also be weighed when considering radiotherapy. In fact, local control should be a concern in cases with risk factors for local recurrence such as disease growth pattern, parametrial involvement, and/or marginal status. 

**Figure 2:**
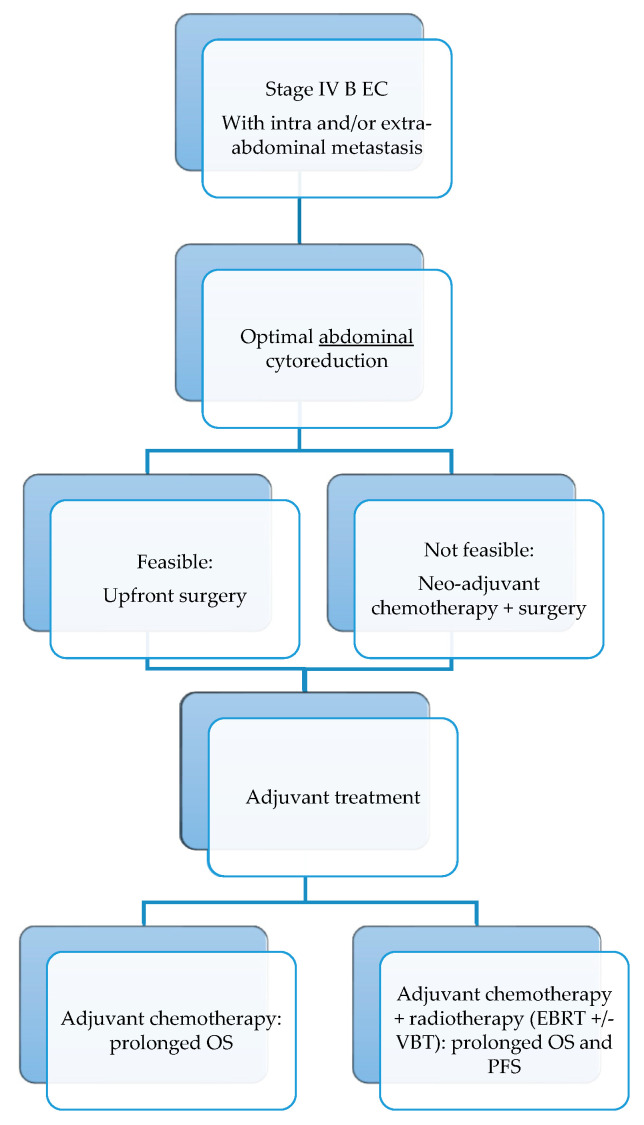
Algorithm proposition for optimal stage IVB endometrial cancer treatment based on the current evidence. EC endometrial cancer; OS overall survival; PFS progression-free survival; EBRT external-beam radiotherapy; VBT vaginal brachytherapy.

An algorithm proposition, based on these observations, is shown in Figure 2.

In 2023, a new FIGO staging system for EC was revised, introducing further stratification of patients with confined pelvic carcinosis (stage IIIB), intra-abdominal metastasis (stage IVB), and extra-abdominal disease spread (stage IVC) [45]. Adopting this novel classification would help to stratify patient risk and regulate the optimal treatment strategy.

Finally, molecular classification is now a cornerstone in EC management, and interesting implications are expected from the ongoing RAINBO trial [5] also in the set of advanced stage disease. Novel evidence on the impact of molecular aspects also in this context may help the better discrimination of prognostic factors in this population, surpassing the constraints linked to conventional pathological factors like histology. Furthermore, several phase III trials are ongoing, investigating the role of immunotherapy as a first-line treatment in advanced-stage patients with microsatellite instability, either in combination with standard chemotherapy or as a monotherapy. Moreover, other potential targeted therapies such as PARP inhibitors in patients with homologous DNA recombination deficiency, trastuzumab in HER2-positive patients, and Selinexor in p53 wild-type patients are being explored for advanced-stage and recurrent disease.

## 5. Conclusions

FIGO stage IVB EC with intra- and/or extra-abdominal spread is a rare entity, and the available data from the literature do not provide enough evidence to draw strong recommendations on the optimal treatment algorithm. Based on the most up-to-date literature available, cytoreductive surgery with complete removal of macroscopic residual tumor is unanimously recognized as a good prognostic factor, NACT should be considered as a feasible option in cases of patients not eligible for optimal cytoreduction, and multimodality adjuvant therapy may be beneficial even in the presence of disease spread beyond the pelvis.

Nevertheless, these considerations are derived from retrospective studies, with a low level of evidence according to the GRADE system. Further studies would be necessary to formulate stronger recommendations [14].

## Supplementary Materials

The following supporting information can be downloaded at: https://www.mdpi.com/article/10.3390/cancers15215123/s1, Table S1. Primary surgery in stage IVB EC. EC endometrial cancer; EBRT external beam radiotherapy; OC ovarian cancer; OS overall survival; PFS progression free survival; PS performance status; RT residual tumor; VBT vaginal brachitherapy. Table S2. Neoadjuvant chemotherapy in stage IVB EC. EC endometrial cancer; IDS interval debulking surgery; ITT intention to treat; NA not assessed; NACT neoadjuvant chemotherapy; OC ovarian cancer; OS overall survival; PFS progression free survival;PP per-protocol; PS performance status; RT residual tumor; Table S3. Adjuvant treatment in stage IVB EC. EC endometrial cancer; NA not assessed; OS overall survival; PFS progression free survival; EBRT external-beam radiotherapy; VBT vaginal brachytherapy.
