# Supplementary material for: Optimal Management for Stage IVB Endometrial Cancer: A Systematic Review
Source: Cancers (Basel). 2023 Oct 24;15(21):5123. doi: 10.3390/cancers15215123 (PMC10649928; doi:10.3390/cancers15215123)
Supplement: Supplementary file 1 [file cancers-15-05123-s001.zip › cancers-2626965-supplementary.pdf]

Table S1 Primary surgery in stage IVB EC. *EC endometrial cancer; EBRT external beam radiotherapy; OC ovarian cancer; OS overall survival; PFS progression free survival; PS performance status; RT residual tumor; VBT vaginal brachitherapy.*

**Author**	**Year**	**N of patients**	**Type of study**	**Inclusion criteria**	**Hystotype**	**N;%**	**Localization of metastasis**	**N; %**	**Residual tumor**	**N;%**	**Adjuvant treatment**		**Main results/Survival**	**Main findings**
Bristow et al.	2000	65	Retrospective study	Surgically treated stage IVB endometrial cancer patients	Endometrioid<br>Serouos<br>Others	22; 33.8<br>21; 32.4<br>22; 33.8	Intra-abdominal metastasis<br>Extra-abdominal metastasis	56; 86.2<br>9; 13.8	Complete cytoreduction<br>Optimal cytoreduction (≤1 cm)<br>Suboptimal cytoreduction	NA<br>36; 55.4<br>29; 44.6	Chemo+radiotherapy<br>Only radiotherapy<br>Only chemotherapy	14; 21.5<br>11; 16.9<br>27; 41.6	OS 34.3 months for RT ≤ 1 cm<br><br>OS 11 months for RT > 1cm	Survival outcomes are influenced by the extent of RT following cytoreductive surgery, the patient's age, and their PS. Additionally, in specific cases, a combination of postoperative chemotherapy and radiation therapy may offer potential therapeutic advantages.
Ayhan et al.	2002	37	Retrospective study	Surgically treated stage IVB endometrial cancer patients	Endometrioid<br>Others	29; 78.4<br>8; 21.6	Intra-abdominal metastasis<br>Extra-abdominal metastasis	31; 83.8<br>6; 16.2	Complete cytoreduction<br>Optimal cytoreduction (≤1 cm)<br>Suboptimal cytoreduction	12; 32.4<br>10; 27<br>15; 55.5	Chemo+radiotherapy<br>Only radiotherapy<br>Only chemotherapy	10; 33.3<br>10; 33.3<br>10; 33.3	OS 48 months for RT 0<br><br>OS 25 months for RT ≤ 1 cm<br><br>Median OS 15 months	The absence of extra-abdominal metastasis, optimal cytoreduction, and multimodality adjuvant treatment are significant factors for improved survival outcomes.
Haight et al	2023	88<br><br>63 primary surgery<br><br>25 NACT	Retrospective study	Surgically treated stage IVB endometrial cancer patients. The 63,6 % of patients were not suspected as stage IVB prior to surgery.	Endometrioid<br>Carcinosarcom<br>Clear cell<br>Dedifferentiated/undifferentiated<br>Mixed<br>Serous<br>High grade ESS	35; 398<br>12; 13.6<br>1; 1.1<br>4; 4.5<br>7; 8<br>28; 31.8<br>1; 1.1	Intra-abdominal metastasis<br>Extra-abdominal metastasis	63; 71.6<br>25; 28.4	Primary surgery:<br><br>Complete cytoreduction<br>Optimal cytoreduction (≤1 cm)<br>Suboptimal cytoreduction	31; 49.2<br>20; 31.7<br>12; 19.1	None<br>EBRT<br>Chemotherapy<br>Chemotherapy + EBRT<br>Chemotherapy + EBRT + VBT	11; 12.5<br>1; 1.1<br>59; 67<br>11; 12.5<br>6; 6.8	Median OS 38 months<br><br>No significant OS difference between optimal and complete cytoreduction.<br><br>Trends in improved OS were noted for patients who had fewer metastatic lesions and sub-centimeter disease, but these were not statistically significant.	Patients diagnosed with stage IVB endometrial cancer (2009 FIGO criteria) comprise a heterogenous cohort of disease. The distribution of metastases, quantification of tumor burden, and degree of cytoreduction are associated with prognosis.

Lee et al	2014	48	Retrospective study	Stage IVB serous EC, with pathologic evidence of intra-abdominal spread.	Serous 48; 100	Intraabdominal metastasis 48; 100<br>Extra-abdominal metastasis 0	Complete cytoreduction 22; 46<br>Optimal cytoreduction (≤1 cm) 14; 29<br>Suboptimal cytoreduction 12; 25	None 8; 17<br>chemotherapy 19; 40<br>chemotherapy and RT 16; 33<br>RT alone 5; 10	OS 26.5 months for RT ≤ 1 cm<br><br>OS 12.6 months for RT > 1 cm	Optimal cytoreduction and chemotherapy are the strongest determinants of long-term survival. Combined chemotherapy and RT were associated with an improvement in PFS and OS.
Eto et al	2012	248	Multicentre, retrospective study	Clinical or surgical stage IVB endometrial cancer patients, undergoing primary surgery.	Endometrioid 149; 61<br>Serous 43; 17<br>Clear cell 15; 6<br>Carcinosarcoma 23; 9<br>Other 18; 7	Intra-abdominal alone 155; 62<br>Extra-abdominal alone 57; 23<br>Both 36; 15	Postoperative residual disease None 62; 25<br>≤1 cm 63; 25<br>>1 cm 12; 50<br><br>Intra-abdominal residual disease<br>None 101; 41<br>≤1 cm 52; 21<br>>1 cm 95; 38<br><br>Extra-abdominal residual disease<br>None 168; 67<br>≤1 cm 24; 10<br>>1 cm 56; 23	None 28; 11<br>chemotherapy 185; 75<br>radiotherapy 11; 4<br>chemo+radiotherapy 24; 10	Postoperative residual disease<br>OS 48 months for RT0<br>OS 25 months for RT ≤ 1 cm<br>OS 17 months for RT > 1cm<br><br>Intra-abdominal residual disease<br>OS 48 months for RT0<br>OS 23 months for RT ≤ 1 cm<br>OS 14 months for RT > 1cm<br><br>Extra-abdominal residual disease<br>OS 26 months for RT0<br>OS 38 months for RT ≤ 1 cm<br>OS 21 months for RT > 1cm	PS, histology/ grade, postoperative treatment, and intra-abdominal residual disease are independent predictors of survival in patients with stage IVb EC who underwent primary cytoreductive surgery, even in the presence of extra-abdominal metastasis.
Ueda et al	2010	33	Retrospective sudy	Surgical stage IVB endometrial cancer patients, undergoing primary surgery.	Endometrioid 24; 72.7<br>Serous and clear cell 9; 27.3	Intra-abdominal 15; 45.5<br><br>Extra-abdominal 18; 54.5	Residual tumor ≤ 2 cm 20;<br>Residual tumor > 2cm 13;	None 3;<br>Chemotherapy 30;	OS 43 months for RT ≤ 2 cm<br>OS 6 months for RT > 2cm	Cytoreduction should be considered for all stage IVb endometrial cancers whose primary tumors are resectable, even those with extra-abdominal metastasis.
Landrum et al	2009	55 EC patients<br><br>110 OC patients	Case-control retrospective study	Surgical stage IVB endometrial cancer patients with only intra-abdominal disease,	EC<br><br>Serous 29; 53<br>Endometrioid 24; 43<br>clear cell 2; 4<br><br>OC<br>Serous 86; 80	Intra-abdominal metastasis 55; 100<br><br>Extra-abdominal metastasis 0	EC<br><br>Complete cytoreduction NA<br>Optimal cytoreduction (≤1 cm) 48; 87<br>Suboptimal cytoreduction 7; 13<br><br>OC NA	EC<br><br>Chemotherapy 33; 60<br>Chemo-radiation 14; 25<br>Radiation 8; 15<br><br>OC	Two-year OS for the entire cohort: 52 vs. 76% for EC vs. OC.<br><br>For optimal cytoreduction,	The current paradigm of treatment for advanced EC including aggressive cytoreduction followed by

				undergoing primary surgery, compared with stage IIIC OC patients.	endometrioid clear cell	14; 13<br>1;1		Complete cytoreduction<br>Optimal cytoreduction (≤1 cm)<br>Suboptimal cytoreduction	99; 90<br>11; 10	Chemotherapy	110; 100	OS at 2 years: 57 vs. 82%.<br><br>For suboptimal EC vs. OC patients: 33% vs. 66%.<br><br>Median PFS was 13 months vs. 20 months for all EC and OC patients.	platinum-based chemotherapy provides the most effective strategy for primary therapy at present. However, compared to OC, prognosis remains poor.		

Table S2 Neoadjuvant chemotherapy in stage IVB EC. EC endometrial cancer; IDS interval debulking surgery; ITT intention to treat; NA not assessed; NACT neoadjuvant chemotherapy; OC ovarian cancer; OS overall survival; PFS progression free survival;PP per-protocol; PS performance status; RT residual tumor;.

**Author**	**Year**	**N of patients**	**Type of study**	**Inclusion criteria**	**Primary treatment**	**n;%**	**Hystotype**	**N;%**	**Localization of metastasis**	**N; %**	**Residual tumor**	**N;%**	**Adjuvant treatment**		**Main results/ Survival**	**Main findings**
Vandenput et al	2009	30	Retrospective study	Stage IVB EC with transperitoneal spread ad laparoscopy	NACT, 3-4 cycles	30; 100	Endometriod<br>Serous<br>Clear cell	2; 6.7<br>27; 90<br>1; 3.3	Intra-abdominal metastasis<br><br>Extra-abdominal metastasis	30;100<br><br>0	IDS<br>No surgery for disease progression<br><br>Complete cytoreduction<br>Optimal cytoreduction (≤1 cm)	24; 80<br>6; 20<br><br>22; 92<br>2; 8	NA		PFS 13 months<br><br>OS 23 months<br><br>OS in inoperable patients 12 months<br><br>Postoperative complications: 17%	Use of NACT results in hight complete cytoreduction rate and low postoperative morbidity.
Nakanishi et al	2023	51	Multicentre, retrospective study	Stage IVB EC at preoperative imaging (CT, MRI, PET)	NACT, 3 cycles	51; 100	Endometrioid<br>Serous<br>Clear cell<br>Adenosquamous<br>Other	34; 69.4<br>9; 18.4<br>2; 4.1<br>1; 2<br>3; 6.1	Intra-abdominal metastasis<br><br>Extra-abdominal metastasis	33; 64.7<br><br>18; 35.3	IDS<br>No surgery for disease progression<br><br>Complete cytoreduction<br>Residual disease (no diameter specified)	33; 65<br>18; 35<br><br>21; 64<br>12; 36	Adjuvant chemotherapy	29; 88	Median overall survival 23.2 months	Neoadjuvant chemotherapy followed by debulking surgery is a feasible and acceptable treatment for stage IVB EC.
Eto et al	2013	426	Multicentre, retrospective study	Clinical and surgical stage IVB EC<br>(n 130 without clinical evidence of stage IVB).	NACT<br><br>Primary surgery<br><br>Palliative care	125; 29.3<br><br>279; 65.5<br><br>22; 5.16	Endometrioid<br>Non-endometrioid	245; 58<br>181; 42	Intra-abdominal metastasis<br><br>Extra-abdominal metastasis	197; 46.2<br><br>229; 53.8	IDS<br><br>Complete cytoreduction<br>Optimal cytoreduction (≤1 cm)<br>Suboptimal cytoreduction<br><br>Primary surgery<br><br>Complete cytoreduction<br>Optimal cytoreduction (≤1 cm)<br>Suboptimal cytoreduction	59; 47<br><br>19; 32<br>15; 25<br>25; 43<br><br>279<br><br>61; 22<br>65; 23<br>153; 55	IDS<br><br>Chemotherapy or Radiotherapy<br><br>Primary surgery<br><br>Chemotherapy<br>Radiotherapy<br>Chemo+radiotherapy	59<br><br>52; 88<br><br>279<br><br>207; 74<br>41; 14.7<br>31; 11.3	OS primary surgery group 21 months<br><br>OS NACT+IDS 21 months<br><br>OS NACT without IDS 7 months	Preoperative chemotherapy may be a useful treatment choice for patients with stage IVb EMCA who are not suitable for primary surgery.

Tobias et al	2020	4890 stage IV<br>3922 stage IVB	Cohort study on National Cancer Database	Stage IV EC < 70 yo	NACT 952; 19.5<br>Primary surgery 3938; 80.5	Endometrioid 1337; 27.3<br>Serous 1077; 22<br>Non-endometrioid 2476; 50.6	NA	NA	NA	In the ITT analysis of all patients, survival was superior for women treated with NACT for the first 3 months after diagnosis. In the PP analysis, mortality was lower for the NACT group until the survival curves crossed after approximately 8 months.	Women treated with PDS are at increased risk of early death but have a more favorable long-term prognosis.
Jooya et al	2023	5505	Retrospective analysis on SEER database	Stage IVB EC receiving primary chemotherapy or primary surgery.	NACT 930; 16.9<br>Primary surgery 3052; 55.4<br>Chemotherapy only 1523; 27.7	Endometrioid 1608; 29.2<br>Serous 1671; 30.3<br>Others 2226; 40.4	Intra-abdominal metastasis 3558; 64.6<br>Extra-abdominal metastasis 1947; 35.4	NA	NA	The median OS was 26 months for the primary surgery group, 25 months for the NACT-surg group, and 9 months for the CT alone group, respectively	NACT-based approach was overall not associated with a difference in OS compared to a primary surgical approach. However, the NACT+IDSgroup showed improved survival outcomes in presence of distant-organ metastatic extent.
Zhang et al	2023	1978	Propensity match analysis on SEER database	Stage IVB EC patients, receiving surgery and chemotheraoy vs. chemotherapy alone.	Cancer directed surgery+chemo 989; 50<br>Chemotherapy alone 989; 50	Endometrioid 503; 25.4<br>Non-endometrioid 1475; 74.6	Intra-abdominal metastasis 928; 46.9<br>Extra-abdominal metastasis 1050; 53.1	NA	NA	Median overall survival times were 21 and 8 months in the cancer-directed surgery group and non-surgery group, respectively.	Surgical treatment can prolong the OS of patients, even if only primary site resection is performed. Surgery should be more aggressive in patients aged < 60 years, and with lesions confined in the pelvis.

Kanno et al	2023	67	Retrospective study	Stage IVB EC with distant metastasis.	NACT 27; 40.3<br><br>Primary surgery 23; 34.3<br><br>Chemotherapy alone 17; 25.4	Endometrioid 13; 19.4<br><br>Non-endometrioid 54; 80.6	Intra-abdominal metastasis 34; 51<br><br>Extra-abdominal metastasis 37; 100	IDS 27<br><br>Complete cytoreduction 11; 40.7<br>Optimal cytoreduction (≤1 cm) 16; 59.3<br><br>Primary surgery 23<br><br>Complete cytoreduction 16; 69.6<br>Optimal cytoreduction (≤1 cm) 7; 30.4	NA	OS for RT 0 27 months<br><br>OS for optimal cytoreduction 12 months<br><br>OS of patients receiving chemotherapy alone 7 months.<br><br>OS 44 months in RT0 primary surgery group, vs 12 months in RT 0 patients the NACT group	Complete cytoreduction could improve survival, even in patients with EC and distant metastases. Regardless of whether a case is primary surgery or IDS, surgery should be performed with the aim of achieving intra-abdominal RT0.
Wilkinson-Ryan et al	2015	44	Retrospective study	Stage IVB EC patients with serous histology	NACT 10; 22.7<br><br>Primary surgery 34; 77.3	Serous 26; 59<br><br>Mixed (serous > 50%) 18; 41	NA	IDS 10<br><br>Complete cytoreduction 7; 70<br>Optimal cytoreduction (≤1 cm) 3; 30<br><br>Primary surgery 34<br><br>Complete cytoreduction 11; 32.3<br>Optimal cytoreduction (≤1 cm) 17; 50<br>Suboptimal cytoreduction 6; 17.7	NA	There was no difference in median PFS (10.4 versus 12.0 months) or OS (17.3 versus 20.7 months) between the NACT and primary surgery groups.	Significant differences were founf in operative time and day of discharge in favor of NACT without appearing to significantly worsen prognosis.
Bogani et al	2019	34	Propensity-match analysis	Stage IVB serous EC for peritoneal dissemination, receiving NACT+IDS vs. primary surgery + chemotherapy	NACT 15; 44.1<br><br>Primary surgery 19; 55.9<br><br>(of these, 15 patients were selected for propensity match analysis)	Serous 34; 100	Intra-abdominal metastasis 34; 100	IDS 15; 50<br><br>Complete cytoreduction 14; 93.3<br>Optimal cytoreduction (≤1 cm) 1; 6.7<br><br>Primary surgery 15; 50<br><br>Complete cytoreduction 13; 86.7<br>Optimal cytoreduction (≤1 cm) 2; 13.3	Chemotherapy 30; 100	Median PFS was 12.0 vs 15.3 months in the NACT vs primary surgery group. Median OS was 16.7 vs 18.0 months respectively	NACT may be a valuable treatment modality for patients with unresectable stage IVB serous EC.

Unsal et al	2022	42	Multicentre, retrospective study	Stage IVB serous EC patients receiving NACT+IDS or primary surgery, with only intra-abdominal disease.	NACT<br><br>Primary surgery	10; 23.8<br><br>32; 76.2	Serous	42; 100	Intra-abdominal metastasis	42; 100	IDS<br><br>Complete cytoreduction<br>Optimal (≤1 cm) and suboptimal cytoreduction<br><br>Primary surgery<br><br>Complete cytoreduction<br>Optimal (≤1 cm) and suboptimal cytoreduction	10<br><br>8; 80<br><br>2; 20<br><br>32<br><br>26; 81<br><br>6; 19	NA		Study group had a 2-year PFS of 32% and 2-year OS of 73%. 5-year PFS was 25% and 5-year OS was 32%.	Receiving NACT did not affect survival outcomes in stage IVB EC patients with intra-abdominal disease.

Table S3 Adjuvant treatment in stage IVB EC. EC endometrial cancer; NA not assessed; OS overall survival; PFS progression free survival.

Author	Year	N of patients	Type of study	Inclusion criteria	Hystotype	N;%	Localization of metastasis	N; %	Adjuvant treatment		Main results/Survival	Main findings
Ayhan et al	2002	37	Retrospective study	Surgically treated stage IVB endometrial cancer patients	Endometrioid<br>Others	29; 78.4<br>8; 21.6	Intra-abdominal metastasis<br>Extra-abdominal metastasis	31; 83.8<br>6; 16.2	Chemo+radiotherapy<br>Only radiotherapy<br>Only chemotherapy	10; 33.3<br><br>10; 33.3<br>10; 33.3	Chemo+radiotherapy was associated with a median OS of 54 monts.<br><br>Median OS in case of only radiotherapy was 15 months, and 13 months for chemotherapy alone.	Concomitant chemo and radiotherapy provided the best survival benefit in stage IVB surgically treated EC patients.
Cirik et al	2016	65	Retrospective study	Stage IVB EC patients with only intra-abdominal metastasis, undergoing primary surgery.	Endometrioid<br>Serous<br>Clear cell<br>Undifferentiated<br>Mixed	38; 58.5<br>14; 21.5<br>6; 9.2<br>5; 7.7<br>2; 3.1	Intra-abdominal metastasis	65; 100	Only radiotherapy<br>Only chemotherapy<br>Chemo+radiotherapy<br>Other	16; 24.6<br>31: 47.7<br>4; 6.1<br>14; 21.6	Two-years PFS in case of adjuvant chemotherapy vs. radiotherapy: 34.9 vs 11.1.<br><br>Two-years OS in case of adjuvant chemotherapy vs. radiotherapy: 44.5 vs 11.	Significant imrpovement in both PFS and OS by using systemic chemotherapy instead of only radiotherapy was shown in stage IVB patients with intra-abdominal disease.
Barrington et al.	2022	17890 stage IV<br><br>16133 stage IVB	Analysis in national cancer registries (NCDB and SEER)	Stage IV EC	Endometrioid<br><br>Serous<br><br>Others	4185; 32.7<br>4426; 34.6<br>4185; 32.7	NA		Stage IVB<br><br>Chemotherapy alone<br><br>Chemo+EBRT<br><br>Chemo+VBT<br><br>Chemo+EBRT+VBT	16133<br><br>12795; 79.3<br>2398; 14.9<br>758; 4.7<br><br>182; 1.1	In both databases, use of any radiation in addition to chemotherapy was associated with improved OS.<br>The addition of radiation to chemotherapy was associated with improved OS in all histologies except clear cell carcinoma.	Multimodality treatment was associated with improved OS in stage IVA and IVB EC patients.
Haight et al.	2023	88<br><br>63 primary surgery<br><br>25 NACT	Retrospective study	Surgically treated stage IVB endometrial cancer patients. The 63,6 % of patients were not suspected as stage IVB prior to surgery.	Endometrioid<br>Carcinosarcom<br>Clear cell<br>Dedifferentiated/undifferentiated<br>Mixed<br>Serous<br>High grade ESS	35; 39.8<br>12; 13.6<br>1; 1.1<br>4; 4.5<br>7; 8<br>28; 31.8<br>1; 1.1	Intra-abdominal metastasis<br>Extra-abdominal metastasis	63; 71.6<br>25; 28.4	None<br>EBRT<br>Chemotherapy<br>Chemotherapy + EBRT<br>Chemotherapy + EBRT + VBT	11; 12.5<br>1; 1.1<br>59; 67<br>11; 12.5<br>6; 6.8	Multimodal adjuvant treatment improved PFS,but not OS, even in patients with pelvic confined disease.	This study confirms the lack of known OS benefit for adjuvant radiation in advanced EC extends to those with FIGO stage IVB disease, even when confined to the pelvis.
